# Within the Wards

**Published:** 1888-10-06

**Authors:** 


### Within the Wards.

### LOCKJAW FROM A SPLINTER IN THE PALM.

It is pretty generally known that an injury to the palm of the hand, if of a serious nature, is liable to be followed by lockjaw. It is commonly believed that lockjaw, if once established, is almost invariably fatal. The following case, reported from Vienna, confirms the former current belief, but refutes the latter. At a recent meeting of the Imperial Royal Society of Physicians, Vienna, Dr. v. Eiselsberg gave an account of a case of tetanus (lockjaw) which had been under the care of Professor Billroth. A woman, aged 40, whilst scrubbing the floor, drove a splinter of wood into the palm of her hand. Efforts were made by her husband to extract the splinter, and a portion of it was thus removed. In the course of a week an abscess formed in the hand, which was opened by the medical man in attendance. On the twelfth day after the injury typical symptoms of severe lockjaw set in, and the woman was removed to Professor Billroth's clinic. The symptoms continued for four weeks. The patient then recovered to a considerable extent, and was permitted to leave the hospital. She still, however, suffered from spasmodic contractions of the arm. Two months later a fistulous opening appeared in the palm at the seat of injury, and a discharge of pus followed. A small splinter of wood which had been left behind came away in the discharge. The cause of irritation being thus removed the wound healed, and the patient made a perfect recovery. A couple of instructive experiments were then made by Dr. v. Eiselsberg. He prepared "cultures" from the piece of wood which had come away in the discharge, and which was presumably impregnated with the poisonous matter found in the wound. With one of the "cultures" two rabbits were inoculated at different dates, and the results carefully watched. On the sixth day after inoculation one of the rabbits showed symptoms of tetanus, and died. The second rabbit, which had been inoculated later, also developed the typical characteristics of tetanus, and was shown in that condition to the Royal Society of Physicians. Dr v. Eiselsberg foretold its death in a few days at most. The lecturer called attention to a fact which will probably startle many people, viz., that the pathogenic bacillus of tetanus may be found in common garden mould and in gravel. There is, however, little need for alarm. The practical conclusion to be drawn is to avoid injuring the palm, and to take care to keep wounds absolutely free from dirt both in the palm and elsewhere.

